# Using multiplex nucleic acid amplification tests in the diagnosis and screening for trichomonas vaginalis and *mycoplasma* genitalium

**DOI:** 10.1177/0956462420987415

**Published:** 2021-02-03

**Authors:** James D Woodward, Megan Dalrymple-Hay, Emanuela Pelossi, Raj Patel

**Affiliations:** 1 7423 Southampton Medical School, Southampton, UK; 2 105634 University Hospitals Southampton, Southampton, UK; 3 Solent NHS Trust, Southampton, UK

Dear Editor

Using nucleic acid amplification tests (NAATs) to test for *Chlamydia trachomatis* (CT) and *Neisseria gonorrhoeae* (NG) is commonplace across Europe. These tests are also available for *Trichomonas vaginalis* (TV) and *Mycoplasma genitalium* (MG); however, their routine use is more controversial since both the prevalence and consequence for management is disputed. In our centre, we have recently shifted our service to use the Abbott Alinity® m STI assay to test for CT and NG. One of the consequences of using this platform is that it automatically tests for all four pathogens in every sample (producing an identifiable result only for the tests requested). Only Abbott has access to results after recording, but on request can produce an anonymised report detailing the total number of samples with positive results for each pathogen.

Reviewing this report for the first 3105 samples and comparing it with clinical records, we made the following observations. Firstly, as expected, TV is substantially underdiagnosed with only five of the 52 cases (9.6%) identified by the Alinity® m platform having been diagnosed in clinic (using microscopy). The second result was that MG was the most common of all four pathogens, with 5.8% of samples testing positive compared to 4.9% for CT and 3.7% for NG.

Both TV and MG are recognised as pathogens which rarely present with symptoms.^1,2^ The consequences of untreated asymptomatic infection certainly for MG are likely to be limited. The current UK guidelines for MG recommend against testing except in symptomatic disease, since patients often require rarely used antibiotics to clear infection with possibly limited benefit to themselves.^3^

Our results also support a cautious approach to the introduction of broader TV testing. Previous studies report wide variation in prevalence dependent on local population characteristics.^4^ We would suggest that by increasing TV NAATs testing (based on HIV status, ethnicity and in all women complaining of vaginal discharge), we would significantly close the gap of undiagnosed infection.^4^

Previous studies have reported up to 10% of patients with CT are co-infected with MG.^5^ This would suggest that over 5% of our population have MG without CT. The range of symptoms attributable to MG is broad, and its significance as a potential pathogen is disputed when other causes are present.^3^ We plan, at present, to only consider the limited introduction of MG testing in symptomatic patients and their partners (men with urethral or testicular symptoms and women with abdominal pain, vaginal bleeding and vaginal discharge). We will continue to review the Alinity® m report and will be able to accurately monitor how useful this approach will be.
